# Parents lose less weight than nonparents in an intensive lifestyle intervention

**DOI:** 10.1002/osp4.436

**Published:** 2020-07-22

**Authors:** Carolyn T. Bramante, Rachel L. J. Thornton, Scott J. Pilla, Nisa M. Maruthur, Maya Venkataramani, Jeanne M. Clark

**Affiliations:** ^1^ Division of General Internal Medicine, Department of Medicine University of Minnesota Minneapolis Minnesota USA; ^2^ Division of General Pediatrics and Adolescent Medicine, Department of Pediatrics Johns Hopkins School of Medicine Baltimore Maryland USA; ^3^ Department of Health, Behavior, and Society Johns Hopkins Bloomberg School of Public Health Baltimore Maryland USA; ^4^ Johns Hopkins School of Public Health, Center for Health Equity Baltimore Maryland USA; ^5^ Welch Center for Prevention, Epidemiology, and Clinical Research Johns Hopkins Medical Institutions Baltimore Maryland USA; ^6^ Division of General Internal Medicine, Department of Medicine Johns Hopkins University School of Medicine Baltimore Maryland USA; ^7^ Department of Epidemiology Johns Hopkins Bloomberg School of Public Health Baltimore Maryland USA

**Keywords:** barriers to weight loss, intergenerational obesity, parental obesity, paediatric obesity

## Abstract

**Objective:**

Understand whether parents lose less weight than nonparents in behavioural weight interventions.

**Methods:**

The Look AHEAD (Action for Health in Diabetes) trial randomized adults with Type 2 diabetes and overweight to an intensive lifestyle intervention (ILI) or control (diabetes support and education [DSE]). Participants who reported living with a child under age 18 were designated as ‘parents’ for this analysis. Intention to treat analysis was performed of the effect of the ILI on change in weight at 1 year by parental status. Adherence to attending intervention visits was compared between parents and nonparents. Subgroup analyses were done based on previous subgroup findings in the Look AHEAD study.

**Results:**

Among 4,547 participants, 15% were parents. Parents were younger and more likely to have self‐identified as African American or Hispanic/Latino. Comparing ILI with DSE, parents lost less weight than nonparents (−7.1% vs. −8.3%, *p* = 0.021). African American female parents lost 4% body weight compared with 7% in African American female nonparents (*p* = 0.01).

**Conclusions:**

In a randomized trial, parents lost less weight than nonparents, and this difference was largest for African American women. These findings suggest parents face unique challenges achieving weight loss; more research is needed to understand and optimize interventions for parents.

## 1 INTRODUCTION

Individuals with obesity have greater cardiovascular (CV) disease, CV mortality, cancer and all‐cause mortality, and obesity during childhood strongly predicts obesity in adulthood.^1^, ^2^, ^3^, ^4^, ^5^ Obesity during childhood is associated with lower educational attainment and lower income in adulthood, as well as CV disease and mortality.^1^, ^2^, ^6^, ^7^ Regardless of a child's current weight status, a parent having obesity more than doubles the risk for that child developing obesity in adolescence.^8^, ^9^, ^10^, ^11^ The majority of adults in the United States have overweight or obesity; meaning, parental obesity is a very prevalent risk factor for childhood obesity, and effective weight loss interventions are urgently needed.^12^, ^13^ Adults who take care of children have less time available for healthy lifestyle activities such as adequate sleep, exercise and preparing healthy foods.^14^, ^15^, ^16^ Making healthy behaviour changes is important for achieving weight loss; however, little is known about parents' specific needs for behaviour changes that produce weight loss. In behavioural weight loss interventions, the focus is typically the behaviour change of the individual without assessing or accounting for their family context.^17^, ^18^, ^19^

However, family context may be an important consideration for achieving lifestyle change. Female‐headed households have lower health status than women in other living arrangements, and single adult fathers and custodial grandfathers face high rates of diabetes, obesity and mental health illness compared with national rates.^20^, ^21^ Family‐based treatment of obesity in children that also targets weight loss in the parents has been shown to be effective for weight loss in both children and their parents,^22^, ^23^, ^24^, ^25^, ^26^, ^27^ and interventions for chronic disease management in parents may have spillover effects on adolescents.^28^ Qualitative research among parents with diabetes has shown that their children's dietary preferences tempt them to eat foods that they are supposed to be avoiding.^29^

Despite the potential effects that being a parent may have on adults' health behaviours, literature is lacking comparing the effectiveness of adult weight‐loss interventions between parents and nonparents. The objective was to compare the effectiveness of the Look AHEAD (Action for Health in Diabetes) trial by parenting status. Participants who reported living with a child under age 18 are referred to as ‘parents’, and participants who reported not living with children under age 18 are referred to as ‘nonparents’. It was hypothesized that parents would lose less weight than nonparents and that this decreased response to the intervention will be explained by parents achieving fewer improvements in behaviour change than nonparents. Additional hypotheses were that there would be differences by race, age and gender, between parents and nonparents, because there were differences in these subgroups in the 1‐year weight‐loss outcomes of the Look AHEAD study.^30^ Given the significant differences in obesity prevalence by race,^12^ it is important to examine all factors that could influence weight loss outcomes. It was further hypothesized that the number of children in the home would not affect outcomes in a linear fashion and that differences in outcomes between parents and nonparents would not be fully explained by engagement in the intervention.

## 2 METHODS

### 2.1 Subjects

This study is a secondary analysis of data from the Look AHEAD clinical trial. Look AHEAD enrolled 5,145 adults aged 45–75 years with Type 2 diabetes and a body mass index (BMI) ≥ 25 kg m^−2^ (or ≥27 kg m^−2^ if taking insulin), who were able to complete a maximal exercise test.^31^, ^32^ Exclusion criteria included the following: significant kidney disease, recent CV disease, exercise limitations due to CV disease, cancer requiring treatment and chronic corticosteroid treatment. Enrolment occurred from 2001 to 2004, with 12‐month results collected from 2002 to 2005. Complete descriptions of the study design, eligibility and recruitment have been published and are available online at https://www.niddkrepository.org/static/studies/look-ahead/, and data used were from the publicly available data repository.^31^, ^32^ The complete cohort has been described (The Look AHEAD Research Group. Baseline characteristics of the randomized cohort from the Look AHEAD research study. Diabetes Vasc Dis Res 2006; 3: 202–215 NIH Registration: NIHMS81811). The Look AHEAD cohort was overall 60% female, average age 59 years, 15% African American, 13% Hispanic/Latino and 63% non‐Hispanic White.^30^ The current analysis used the Look AHEAD distributed data set that excludes data from the Southwest Native American study sites due to consent limitations, yielding a sample size of 4,901 participants. The 4,547 participants who had baseline data available were examined regarding the presence of children in the home.

### 2.2 Look AHEAD intervention

Participants were randomized 1:1 to an intensive lifestyle intervention (ILI) or a diabetes support and education (DSE) arm, which was the control arm. Participants in the ILI arm received a behavioural intervention aimed at improving diet and exercise to produce a goal of 7% weight loss of initial body weight.^33^ The intervention included four in‐person meetings per month for the first 6 months and three in‐person meetings per month from 6 to 12 months. The intervention was modelled after the Diabetes Prevention Programme and included a prescribed portion‐controlled diet, including meal replacement shakes and frozen entrees, in order to achieve caloric reduction.^34^ The physical activity recommendations focused on home‐based exercises and working up to 175 min per week of moderate to vigorous physical activity.^34^ The DSE group received three educational sessions on diet and exercise, and one support session during the first year.^35^

### 2.3 Measures assessed

#### 2.3.1 Children living in the home

The predictor of interest was the presence of children living in the home, based on the response to the following question: ‘Do you have any children or stepchildren living with you now?’ (Yes/No); ‘How many are younger than 18 years old?’ The response to this question was used as a surrogate for current parenting status, as the survey did not assess the relationship between the participants and children in home. Only participants who had children younger than 18 years old living in the home were included as parents.

##### Sociodemographic variables

The presence and number of children living in the home was ascertained by questionnaire at study baseline, as were sociodemographic information and medical history. Questionnaires were self‐administered during study visits, with study staff available for assistance. Highest level of education achieved was assessed in four categories (less than high school, high school diploma, BA or master's degree and doctoral or professional degree). Yearly family income was assessed in five categories (<$20,000 to ≥ $80,000).

##### Anthropometric assessments and medical history

Medication use was assessed by study staff with participants instructed to bring in medications for review. Weight and height were measured at baseline and at 12 months using a stadiometer and standardized protocols. Blood pressure and waist circumference were also measured at baseline and 12 months using standardized protocols. Laboratory measurements were performed at baseline and at 12 months.

##### Behavioural assessments and adherence to intervention

Physical activity and dietary intake were assessed using self‐administered standardized questionnaires for patient recall at baseline and 12 months.^32^ Physical activity was assessed by asking the number of times per week the participants exercised to a degree that made them sweat, felt their heart thump or were short of breath. Adherence to the intervention was examined, measured as the percent of expected intervention visits attended in the ILI group (adherence was not ascertained for the DSE group).

#### 2.3.2 Analytic approach

**FIGURE 1:**
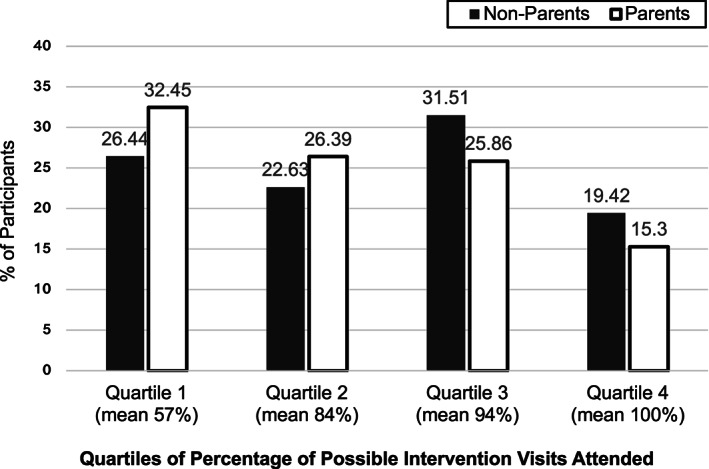
Bar chart that represents the quartiles of percentage of possible intervention visits attended between parents and nonparents. Nonparents are represented by the black bars, and parents are represented by the white bars with black outline. The vertical access represents the percentage of participants who attended each quartile of possible visits (a higher percentage of parents attended the first quartile, and a high percentage of nonparents attended the fourth quartile)

In 2017/2018 differences in participant characteristics at baseline were compared using *χ* ^2^ tests (categorical) or *t* tests (continuous). Logistic regression was used to compare differences in baseline comorbidities by parenting status, adjusted for age. Intention to treat analysis was used to examine the effects of the intervention at 12 months on objectively measured change in weight and CV risk factors, as well as change in self‐reported behaviours (dietary intake and exercise), for parents and nonparents. To determine whether parenting modified these outcomes, linear regression (continuous outcomes) or logistic regression (categorical outcomes) was used, with a multiplicative interaction term for parenting status and treatment arm. Statistical significance was determined by the likelihood ratio test, with *p* ≤ 0.05 considered significant. Regression analyses were performed unadjusted and with multivariable adjustment for the baseline level of the outcome and other baseline variables: age, gender, race, highest level of education, yearly family income, marital status, employment status and BMI. Absolute changes in CV risk factors and behaviour changes were examined. A sensitivity analysis was conducted in which adjusting for number of children in the home was done. Analyses comparing weight loss outcomes for parents versus nonparents were performed among subgroups of age (divided at 54 years), gender and race/ethnicity, because of differences in outcomes in these subgroups found in Look AHEAD.^30^ Adherence was analysed in quartiles: Quartile 1 (0.0%–76.2% visits attended, mean 56.8%), Quartile 2 (78.6%–88.1%, mean 84.0%), Quartile 3 (90.5%–97.6%, mean 94.2%), and Quartile 4 (100% attendance).^30^ The chi‐squared test was performed to test the null hypothesis that parents and nonparents are independent in terms of their distribution into four categories representing quartiles of intervention adherence. The chi‐squared test does not include a reference group (Figure 1).^30^ Adjustment for intervention adherence was added to the regression analysis of the weight outcome after finding a difference in adherence among parents and nonparents to examine whether this was a mediator of the effect of parenting status on outcomes of the intervention.

All analyses were conducted using Stata 14 (StataCorp). The analyses performed herein were not conducted at the Look AHEAD data coordinating centre. This does not represent the work of the Look AHEAD study group.

## 3 RESULTS

Among the 4,547 participants, 699 (15%) were parents, defined as having a child or stepchild younger than age 18 living in the home. Parents were younger than nonparents (54.1 vs. 59.9 years, *p* < 0.001) and more likely to be African American (20.2% vs. 15.4%, *p* < 0.001) or Hispanic/Latino (19.3% vs. 12.5%, *p* < 0.001), (Table 1). Parents were more likely to have a professional degree (6.7% vs. 5.0%, *p* = 0.01) or no high school education (8.5% vs. 5.7%, *p* = 0.01). Yearly household income was higher among parents than nonparents, with 38% of parents reporting >$80,000/year in household income, compared with 25% of nonparents (*p* < 0.001). Parents were also more likely to be employed than nonparents. There was no difference in baseline BMI by parenting status. There was also no difference in age‐adjusted baseline comorbidities between parents and nonparents (Table 1).

**TABLE 1:** Baseline characteristics

Characteristics	Participants with children living in the home	Participants with no children living in the home	*p* value^a^
*n*	699	3,848	
Female gender	405 (57.9)	2,246 (58.4)	0.833
Age, years (mean ± SD)	54.1 ± 6.4	59.9 ± 6.5	**<0.001**
Married^b^	528 (75.5)	2,562 (66.6)	**<0.001**
Unemployed	116 (16.6)	838 (21.8)	**0.002**
BMI (kg m^−2^, mean ± SD)	36.2 ± 6.1	35.9 ± 5.8	0.115
Race/ethnicity			
White, non‐Hispanic	391 (55.9)	2,644 (68.7)	**<0.001**
African American	141 (20.2)	592 (15.4)	
Hispanic/Latino	135 (19.3)	482 (12.5)	
Other	32 (4.6)	130 (3.4)	
Number of children in home			
1	457 (65.4)	n/a	n/a
2	177 (25.3)	n/a	
3+	65 (9.3)	n/a	
Highest level of education			
Doctorate or Professional	47 (6.7)	194 (5.0)	**0.014**
BA or Masters	241 (34.5)	1,446 (37.6)	
High school diploma	337 (48.3)	1917 (49.8)	
Less than high school	59 (8.5)	218 (5.7)	
Other	14 (2.0)	73 (1.9)	
Yearly family income			
>$80,000	268 (38.3)	978 (25.4)	**<0.001**
$60,000–$80,000	101 (14.5)	571 (14.8)	
$40,000–$60,000	107 (15.3)	743 (19.3)	
$20,000–$40,000	112 (16.0)	763 (19.8)	
<$20,000	66 (9.4)	409 (10.6)	
Missing	45 (6.4)	384 (10.0)	
Health Status			
Hypertension^c^	550 (78.7)	3,251 (84.5)	0.76^c^
Dyslipidemia^c^	457 (65.4)	2,754 (71.6)	0.23^c^
Cardiovascular disease^c^	74 (10.6)	571 (14.8)	0.31^c^
Depression^c^	158 (22.6)	796 (20.7)	0.53^c^
Proportion in intervention arm	383 (54.8)	1899 (49.4)	**0.008**

*Note*: Values are mean ± SD or frequency (% of column). Boldface indicates statistical significance (*p* < 0.05).

Abbreviation: BMI, body mass index.

^a^ By *χ* ^2^ test (categorical) or *t* test (continuous).

^b^ Includes ‘living in a marriage‐like relationship’.

^c^ Includes adjustment for age. Boldface indicates statistical significance (*p* < 0.05).

The effect of the intervention (treatment arm minus control arm) on weight and other CV risk factors by parenting status is shown in Table 2. In response to the intervention, parents lost less weight than nonparents, (−7.1% vs. −8.3% of initial body weight, *p* = 0.02), which represents a 14.5% difference in weight loss between parents and nonparents. Parents had less improvement in waist circumference (−5.7 vs. −7.1 cm, *p* = 0.01) compare to non‐parents. The interactions between parenting status and the intervention effect on other CV risk factors were not significant. A greater percentage of nonparents were in the top two quartiles of attendance at possible intervention visits, and a greater percentage of parents were in the lower two quartiles of attendance at possible intervention visits (*p* < 0.01, Figure 1). When adjusting for attendance, there was no difference in weight loss between parents and nonparents.

**TABLE 2:** Unadjusted and adjusted effects of the intervention on 12‐month changes in clinical parameters and self‐reported behavioural outcomes

Outcome	Unadjusted effect of intervention (ILI‐DSE)			Adjusted effect of intervention (ILI‐DSE)		
	Nonparents	Parents	*p* interaction^a^	Nonparents	Parents	*p* interaction^a^
	*n* = 3,848	*n* = 699		*n* = 3,844^b^	*n* = 696^b^	
Cardiovascular disease risk factors (absolute change unless otherwise indicated)						
Weight, % change of baseline weight	−8.2	−7.2	0.04	−8.3	−7.1	**0.02**
Waist circumference, in cm	−7.0	−6.1	0.14	−7.1	−5.7	**0.01**
HbA1c, percent	−0.52	−0.50	0.82	−0.53	−0.55	0.78
Systolic BP, in mmHg	−3.9	−3.6	0.82	−4.5	−3.9	0.59
Diastolic BP, in mmHg	−1.1	−1.8	0.32	−1.4	−2.0	0.38
LDL cholesterol, in mg dl^−1^	0.68	−0.06	0.77	0.46	−0.90	0.53
HDL cholesterol, in mg dl^−1^	2.2	2.0	0.78	2.12	2.20	0.91
Triglycerides, in mg dl^−1^	−17.1	−14.2	0.71	−17.7	−6.1	0.06
Stopped using diabetes medications^c^	7.41	6.61	0.91	7.4	6.7	0.99
Stopped blood pressure medications^c^	0.56	−1.15	0.46	0.5	−1.1	0.53
Stopped lipid‐lowering medication^c^	1.83	−1.09	0.52	1.8	−0.7	0.52
Resolution of metabolic syndrome^d^	8.96	2.85	0.07	9.0	2.9	0.12
Self‐reported behaviours, change between baseline to 12‐month surveys						
Times exercised per week	1.48	1.07	0.24	1.47	1.07	0.17
Total calories consumed per day	−10.5	10.6	0.79	−42.0	21.6	0.31
Servings of sweets/fats per day	−0.53	−0.41	0.47	−0.59	−0.37	0.06
Servings of vegetables per day	0.48	0.33	0.37	0.45	0.41	0.78
Servings of fruits per day	0.33	0.26	0.62	0.32	0.29	0.82
Times eating out per week	−0.37	−0.48	0.59	−0.41	−0.46	0.79

*Note*: Adjusted for age, gender, race, education, income, marital status, employment status, BMI and baseline values. Boldface indicates statistical significance (*p* < 0.05).

Abbreviations: DSE, diabetes support and education; HDL, high‐density lipoprotein; ILI, intensive lifestyle intervention; LDL, low‐density lipoprotein.

^a^ Interaction between having children in the home and treatment arm by ANCOVA using linear regression (continuous outcome) or logistic regression (binary outcome).

^b^ The total is lower than for the unadjusted because some participants were missing the adjusting variables.

^c^ Among those using the medication at baseline, the percent that stopped using medication (treatment–control).

^d^ Among those with metabolic syndrome at baseline, the percent who had resolution of their metabolic syndrome (treatment–control).

There were no statistically significant differences in changes in self‐reported behavioural outcomes (Table 2). There was a trend towards parents having less improvement in servings of sweets and fats consumed per day compared with nonparents (−0.36 vs. −0.59 servings, *p* = 0.06). Parents also had less improvement in number of times exercised per week (1.07 vs. 1.47 times, *p* = 0.17) and less improvement in number of calories consumed per day (22 vs. −42 calories, *p* = 0.31), but these were not statistically significant.

**FIGURE 2:**
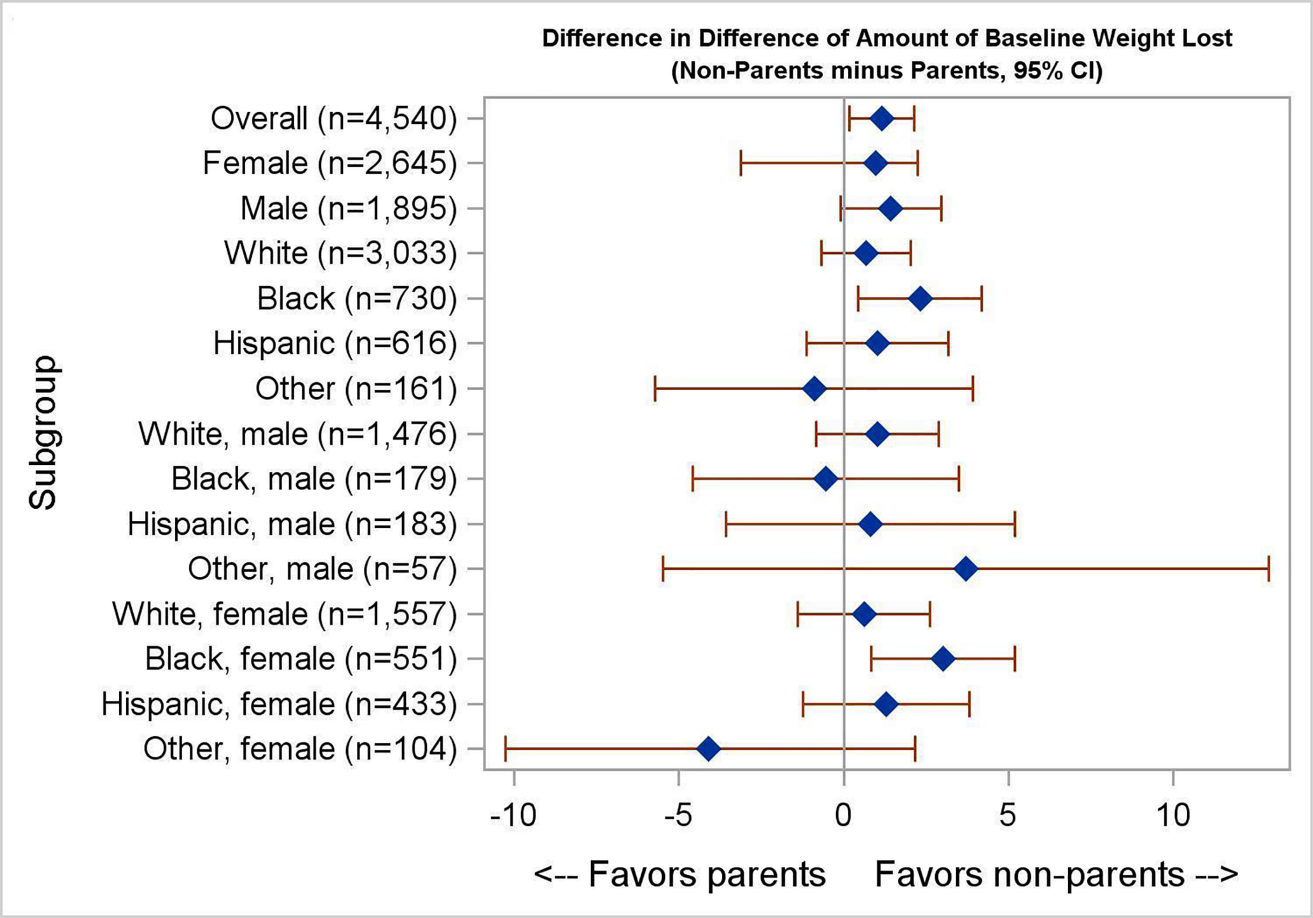
Forest plot of the difference in difference of amount of baseline weight loss between parents and nonparents. Each diamond represents the mean difference in weight loss, nonparents minus parents, for that subgroup. The right side of the central line represents subgroups in which nonparents lost more weight than parents. The left side of the central line represents subgroups in which parents lost more weight than nonparents. The error bars represent 95% confidence intervals for the mean difference in difference values. The *x*‐axis indicates percent of baseline weight loss

In subgroup analyses examining adjusted changes in weight (Table 3, Figure 2), the difference in intervention efficacy between parents and nonparents was most pronounced in African American participants (−4.7% vs. −6.9% weight loss, *p* = 0.02) compared with Hispanic/Latino participants (−6.8% vs. −8.3%, *p* = 0.15) and non‐Hispanic White participants (−8.0% vs. −8.7%, *p* = 0.31). African American parents under age 54 lost less weight than African American parents over 54 years (−3.0% vs. −7.6%, *p* = 0.03). This difference was predominantly driven by African American females (−4.1% vs. −7.0%, *p* = 0.01), especially those aged 54 or younger (−2.0% vs. −8.1%, *p* = 0.01).

**TABLE 3:** Subgroup analysis of adjusted percent baseline weight lost

Subgroup		Adjusted effect of intervention (ILI‐DSE)^a^		
		Nonparents	Parents	*p* interaction^b^
Overall (*n* = 4,540)		−8.3	−7.1	**0.02**
Gender				
Male (*n* = 1,895)		−9.1	−7.7	0.07
Female (*n* = 2,645)		−7.7	−6.8	0.13
Age				
Age <54 years (*n* = 851)		−8.1	−6.7	0.13
Age ≥54 years (*n* = 3,689)		−8.3	−7.3	0.11
Race				
Non‐Hispanic White (*n* = 3,033)		−8.7	−8.0	0.31
African American (*n* = 730)		−6.9	−4.7	**0.02**
Hispanic/Latino (*n* = 616)		−8.3	−6.8	0.15
Other (*n* = 161)		−6.9	−8.0	0.62
Gender and race				
Male	Non‐Hispanic White (*n* = 1,476)	−9.5	−8.6	0.30
	African American (*n* = 179)	−6.7	−7.1	0.79
	Hispanic (*n* = 183)	−7.4	−5.6	0.39
	Other (*n* = 57)	−10.1	−7.0	0.41
Female	Non‐Hispanic White (*n* = 1,557)	−8.0	−7.4	0.56
	African American (*n* = 551)	−7.0	−4.1	**0.01**
	Hispanic/Latino (*n* = 433)	−8.7	−7.1	0.18
	Other (*n* = 104)	−4.8	−9.0	0.14
Age and gender				
Male	<54 years (*n* = 273)	−8.6	−6.6	0.22
	≥54 years (*n* = 2,067	−9.1	−8.6	0.60
Female	<54 years (*n* = 578)	−7.9	−6.9	0.39
	≥54 years (*n* = 2,067)	−7.7	−6.2	0.08
Race and age				
Non‐Hispanic White	<54 years (*n* = 497)	−8.1	−7.9	0.91
	≥54 years (*n* = 2,536)	−8.8	−7.9	0.35
African American	< 54 years (*n* = 168)	−7.6	−3.0	**0.03**
	≥ 54 years (*n* = 562)	−6.6	−5.7	0.37
Hispanic/Latino	< 54 years (*n* = 146)	−6.8	−5.7	0.56
	≥ 54 years (*n* = 470)	−8.3	−7.7	0.67
Other	<54 years (*n* = 40)	−15.4	−9.8	0.15
	≥54 years (*n* = 121)	−5.4	−7.3	0.57

*Note*: Boldface indicates statistical significance (*p* < 0.05).

Abbreviations: BMI, body mass index; DSE, diabetes support and education; ILI, intensive lifestyle intervention.

^a^ Adjusted for age, gender, race, (except for the stratifying variable), education, income, marital status, employment status, BMI and baseline weight.

^b^ Interaction between having children in the home and treatment arm by ANCOVA using linear regression (continuous outcome) or logistic regression (binary outcome).

In sensitivity analysis adjusting for the number of children in the home, the results did not change substantively (Tables 4 and 5). Absolute changes in CV risk factors are shown in Table 6, and changes in behaviours in Table 7. Besides differences in changes in weight and waist circumferences, there were no differences between parents and nonparents in changes in other CV risk factors or behaviours.

**TABLE 4:** Sensitivity analysis including adjustment for number of children in the home for adjusted effects of the intervention

Outcome	Unadjusted effect of intervention (ILI‐DSE)			Adjusted effect of intervention (ILI‐DSE)		
	Nonparents	Parents	*p* interaction^a^	Nonparents	Parents	*p* interaction^a^
	*n* = 3,848	*n* = 699		*n* = 3,844^b^	*n* = 696^b^	
Cardiovascular disease risk factors (absolute change unless otherwise stated)						
Weight, % change of baseline weight	−8.2	−7.2	0.04	−8.3	−7.2	0.02
Waist circumference, in cm	−7.0	−6.1	0.14	−7.1	−5.7	0.02
HbA1c, in percent	−0.52	−0.50	0.82	−0.53	−0.54	0.85
Systolic BP, in mmHg	−3.9	−3.6	0.82	−4.5	−3.8	0.57
Diastolic BP, in mmHg	−1.1	−1.8	0.32	−1.4	−1.9	0.39
LDL cholesterol, in mg dl^−1^	0.68	−0.06	0.77	0.46	−0.90	0.53
HDL cholesterol, in mg dl^−1^	2.2	2.0	0.78	2.13	2.18	0.92
Triglycerides, in mg dl^−1^	−17.1	−14.2	0.71	−17.7	−5.8	0.06
Stopped diabetes medications^a^, b	7.41	6.61	0.91	7.40	6.69	0.97
Stopped blood pressure medications^b^	0.56	−1.15	0.46	0.056	−0.11	0.55
Stopped lipid‐lowering medication^a^, b	1.83	−1.09	0.52	1.85	−0.68	0.57
Resolution of metabolic syndrome^b^	8.96	2.85	0.07	8.97	2.97	0.12
Self‐reported behavioural change						
Times exercised per week	1.48	1.07	0.24	1.47	1.07	0.17
Total calories consumed per day	−10.5	10.6	0.79	−42.0	21.4	0.31
Servings of sweets/fats per day	−0.53	−0.41	0.47	−0.59	−0.36	0.06
Servings of vegetables per day	0.48	0.33	0.37	0.45	0.41	0.80
Servings of fruits per day	0.33	0.26	0.62	0.32	0.30	0.83
Times eating out per week	−0.37	−0.48	0.59	−0.41	−0.46	0.78

*Note*: Adjusted for age, gender, race, education, income, marital status, number of children, employment status, BMI and baseline values.

Abbreviations: DSE, diabetes support and education; HDL, high‐density lipoprotein; ILI, intensive lifestyle intervention; LDL, low‐density lipoprotein.

^a^ Interaction between having children in the home and treatment arm by ANCOVA using linear regression (continuous outcome) or logistic regression (binary outcome).

^b^ The total is lower than for the unadjusted because some participants were missing the adjusting variables.

^c^ Among those using the medication at baseline, the percent who stopped using medication (treatment–control).

^d^ Among those with metabolic syndrome at baseline, the percent who had resolution of their metabolic syndrome (treatment–control).

**TABLE 5:** Sensitivity analysis including adjustment for number of children in the home on 1‐year weight outcomes

Subgroup		Adjusted effect of intervention on % baseline weight lost (ILI‐DSE)^a^		
		Nonparents	Parents	*p* interaction^b^
Overall (*n* = 4,540)		−8.3	−7.2	0.02
Gender				
Male (*n* = 1,895)		−9.0	−7.7	0.07
Female (*n* = 2,645)		−7.7	−6.8	0.14
Age				
Age <54 years (*n* = 851)		−8.1	−6.8	0.15
Age ≥54 years (*n* = 3,689)		−8.3	−7.3	0.11
Race				
Non‐Hispanic White (*n* = 3,033)		−8.7	−8.0	0.33
African American (*n* = 730)		−6.9	−4.6	0.01
Hispanic/Latino (*n* = 616)		−8.3	−7.3	0.35
Other (*n* = 161)		−6.9	−7.8	0.69
Gender and race				
Male	Non‐Hispanic White (*n* = 1,476)	−9.5	−8.5	0.28
	African American (*n* = 179)	−6.6	−7.1	0.78
	Hispanic (*n* = 183)	−7.3	−6.5	0.70
	Other (*n* = 57)	−10.3	−6.6	0.32
Female	Non‐Hispanic White (*n* = 1,557)	−8.0	−7.4	0.55
	African American (*n* = 551)	−7.0	−4.0	0.01
	Hispanic/Latino (*n* = 433)	−8.7	−7.4	0.30
	Other (*n* = 104)	−4.9	−8.9	0.15
Age and gender				
Male	<54 years (*n* = 273)	−8.6	−6.6	0.20
	54 years (*n* = 1,622)	−9.1	−8.6	0.60
Female	<54 years (*n* = 578)	−7.9	−7.1	0.48
	≥54 years (*n* = 2,067)	−7.7	−6.1	0.08
Race and age				
Non‐Hispanic White	<54 years (*n* = 497)	−8.1	−7.9	0.91
	≥54 years (*n* = 2,536)	−8.8	−8.0	0.41
African American	<54 years (*n* = 168)	−7.5	−2.7	0.02
	≥54 years (*n* = 562)	−6.6	−5.6	0.32
Hispanic/Latino	<54 years (*n* = 146)	−6.9	−6.0	0.63
	≥54 years (*n* = 470)	−8.3	−8.2	0.97
Other	<54 years (*n* = 40)	−15.4	−9.8	0.15
	≥54 years (*n* = 121)	−5.4	−6.8	0.69

Abbreviations: BMI, body mass index; DSE, diabetes support and education; ILI, intensive lifestyle intervention.

^a^ Adjusted for age, gender, race, (except for the stratifying variable), education, income, marital status, number of children, employment status, BMI and baseline weight.

^b^ Interaction between having children in the home and treatment arm by ANCOVA using linear regression (continuous outcome) or logistic regression (binary outcome).

**TABLE 6:** Changes in weight and cardiovascular risk factors after 1 year of intervention

Characteristic	Participants with children in the home			Participants with no children in the home			*p* value interaction^a^
	ILI	DSE	*p* value	ILI	DSE	*p* value	
*n*	383	316		1,899	1,949		
Weight (kg)							
Baseline	102.8 ± 1.0	100.8 ± 1.1	0.196^b^	100.7 ± 0.4	101.2 ± 0.4	0.412^b^	
Year 1	97.0 ± 1.1	99.1 ± 1.1	0.196^b^	91.0 ± 0.4	100.5 ± 0.4	<0.001^b^	
% change	−7.8 ± 0.3	−0.6 ± 0.3	<0.001^b^	−9.0 ± 0.2	−0.7 ± 0.11	<0.001^b^	0.019
Waist circumference							
Baseline (cm)	115.1 ± 0.8	113.2 ± 0.8	0.082^b^	113.6 ± 0.3	114.1 ± 0.3	0.231^b^	
Year 1 (cm)	109.4 ± 0.8	111.7 ± 0.9	0.055^b^	105.3 ± 0.3	113.2 ± 0.3	<0.001^b^	
Change (cm)	−7.3 ± 0.5	−1.2 ± 0.3	<0.001^b^	−7.8 ± 0.2	−0.8 ± 0.2	<0.001^b^	0.008
Diabetes medication use							
Baseline	337 (88.9)	277 (88.2)	0.772^c^	1,625 (86.3)	1,670 (86.7)	0.711	
Year 1	267 (81.9)	250 (89.3)	0.010^c^	1,431 (77.7)	1,594 (87.6)	<0.001	
Stopped use^d^	32 (9.5)	8 (2.9)	0.001^c^	170 (10.5)	51 (3.1)	<0.001	0.995
HbA1c (%)							
Baseline	7.33 ± 0.06	7.43 ± 0.07	0.249^b^	7.21 ± 0.03	7.26 ± 0.03	0.140^b^	
Year 1	6.82 ± 0.07	7.38 ± 0.08	<0.001^b^	6.55 ± 0.02	7.10 ± 0.03	<0.001^b^	
Change	−0.57 ± 0.06	−0.07 ± 0.06	<0.001^b^	−0.66 ± 0.02	−0.14 ± 0.02	<0.001^b^	0.780
HTN^e^ med use, *n* (%)							
Baseline	257 (68.4)	197 (64.0)	0.227^c^	1,393 (73.9)	1,410 (73.6)	0.787^c^	
Year 1	223 (69.5)	185 (66.6)	0.444^c^	1,374 (74.6)	1,371 (75.7)	0.437^c^	
Stopped use^d^	14 (5.5)	13 (6.6)	0.607^c^	74 (5.3)	67 (4.8)	0.497^c^	0.525
Systolic BP (mmHg)							
Baseline	127.3 ± 0.9	128.0 ± 0.9	0.593^b^	128.6 ± 0.4	129.9 ± 0.4	0.014^b^	
Year 1	120.7 ± 0.9	125.7 ± 1.0	<0.001^b^	121.6 ± 0.4	126.9 ± 0.4	<0.001^b^	
Change	−6.6 ± 0.8	−3.1 ± 0.9	0.004^b^	−6.8 ± 0.4	−2.9 ± 0.4	<0.001^b^	0.649
Diastolic BP (mmHg)							
Baseline	71.2 ± 0.5	71.7 ± 0.5	0.499^b^	69.7 ± 0.2	70.3 ± 0.2	0.060^b^	
Year 1	68.7 ± 0.5	70.3 ± 0.6	0.045^b^	66.6 ± 0.2	68.4 ± 0.2	<0.001^b^	
Change	−2.8 ± 0.4	−1.0 ± 0.5	0.007^b^	−3.1 ± 0.2	−2.0 ± 0.2	<0.001^b^	0.366
Lipid‐lowering medication use							
Baseline	163 (43.9)	133 (43.5)	0.902^c^	983 (52.8)	996 (52.3)	0.768^c^	
Year 1	149 (46.1)	132 (47.8)	0.678^c^	1,023 (56.1)	1,094 (61.0)	0.003^c^	
Stopped use^d^	35 (21.5)	30 (22.6)	0.823^c^	170 (17.3)	154 (15.5)	0.271^c^	0.533
LDL cholesterol (mg dl^−1^)							
Baseline	115.0 ± 1.6	115.8 ± 1.8	0.752^b^	111.7 ± 0.7	112.1 ± 0.7	0.692^b^	
Year 1	112.8 ± 1.9	112.5 ± 1.9	0.909^b^	106.4 ± 0.7	106.1 ± 0.8	0.763^b^	
Change	−4.2 ± 1.6	−4.2 ± 1.6	0.979^b^	−5.2 ± 0.7	−5.9 ± 0.7	0.472^b^	0.536
HDL cholesterol (md dl^−1^)							
Baseline	42.7 ± 0.6	43.0 ± 0.7	0.689^b^	43.6 ± 0.3	43.7 ± 0.3	0.738^b^	
Year 1	45.7 ± 0.7	44.1 ± 0.7	0.112^b^	47.3 ± 0.3	45.1 ± 0.3	<0.001^b^	
Change	2.7 ± 0.4	0.7 ± 0.3	<0.001^b^	3.6 ± 0.2	1.4 ± 0.2	<0.001^b^	0.890
Triglycerides (mg dl^−1^)							
Baseline	191.2 ± 6.5	175.1 ± 7.1	0.093^b^	179.7 ± 2.6	180.8 ± 2.6	0.773^b^	
Year 1	170.0 ± 6.7	169.4 ± 6.7	0.950^b^	147.8 ± 2.0	166.4 ± 2.2	<0.001^b^	
Change	−20.6 ± 6.4	−6.4 ± 5.0	0.093^b^	−32.3 ± 2.2	−15.2 ± 2.1	<0.001^b^	0.060
Metabolic syndrome							
Baseline	354 (92.4)	294 (93.0)	0.758^c^	1,768 (93.1)	1829 (93.8)	0.352^c^	
Year 1	277 (84.2)	257 (90.5)	0.020^c^	1,498 (80.1)	1,695 (92.2)	<0.001^c^	
Resolved^d^	51 (14.4)	34 (11.6)	0.286^c^	341 (19.3)	189 (10.3)	<0.001^c^	0.107

Abbreviations: DSE, diabetes support and education; HDL, high‐density lipoprotein; ILI, intensive lifestyle intervention; LDL, low‐density lipoprotein.

^a^ Interaction between having children in the home and treatment arm by ANCOVA using linear regression (continuous outcome) or logistic regression (binary outcome).

^b^ Two‐sample *t* test.

^c^ *χ* ^2^ test

^d^ Among those using medication or with metabolic syndrome at baseline.

^e^ HTN med = Hypertension medication.

**TABLE 7:** Self‐reported behavioural changes in diet and exercise

Characteristic	Participants with children in the home			Participants with no children in the home			*p* value interaction^a^
	ILE	DSE	*p* value^b^	ILI	DSE	*p* value	
*n*, exercise data	179	148		927	939		
Exercise per week^e^							
Baseline	1.41 ± 0.14	1.61 ± 0.17	0.361	1.74 ± 0.07	1.73 ± 0.07	0.877	0.165
Year 1	2.86 ± 0.21	1.93 ± 0.20	0.002	3.27 ± 0.09	1.79 ± 0.08	<0.001	
Change	1.46 ± 0.23	0.39 ± 0.21	<0.001	1.51 ± 0.10	0.03 ± 0.09	<0.001	
*n*, dietary data	256	215		962	991		
Total calories per day^c^							
Baseline	2,103 ± 56	2,122 ± 69	0.823	1951 ± 26	1976 ± 28	0.507	0.293
Year 1	1,779 ± 47	1,755 ± 63	0.764	1,620 ± 19	1,674 ± 25	0.079	
Change	−364 ± 51	−374 ± 61	0.893	−313 ± 25	−302 ± 24	0.762	
Sweets and fats per day							
Baseline	2.18 ± 0.11	2.21 ± 0.14	0.856	2.09 ± 0.05	2.18 ± 0.05	0.256	0.055
Year 1	1.30 ± 0.09	1.72 ± 0.12	0.004	1.08 ± 0.03	1.67 ± 0.05	<0.001	
Change	−0.97 ± 0.12	−0.56 ± 0.12	0.014	−0.99 ± 0.05	−0.46 ± 0.05	<0.001	
Vegetables per day							
Baseline	3.16 ± 0.11	3.02 ± 0.11	0.360	2.88 ± 0.05	2.93 ± 0.05	0.465	0.784
Year 1	3.24 ± 0.12	2.88 ± 0.12	0.039	3.18 ± 0.05	2.78 ± 0.05	<0.001	
Change	0.14 ± 0.11	−0.19 ± 0.10	0.035	0.32 ± 0.05	−0.16 ± 0.05	<0.001	
Fruits per day							
Baseline	1.88 ± 0.09	1.77 ± 0.09	0.393	1.97 ± 0.05	1.98 ± 0.04	0.874	0.818
Year 1	2.12 ± 0.09	1.82 ± 0.09	0.026	2.11 ± 0.04	1.80 ± 0.04	<0.001	
Change	0.16 ± 0.09	−0.10 ± 0.09	0.038	0.14 ± 0.05	−0.19 ± 0.04	<0.001	
Times eat out per week							
Baseline	3.47 ± 0.15	3.59 ± 0.17	0.586	3.35 ± 0.08	3.48 ± 0.08	0.245	0.817
Year 1	2.78 ± 0.16	3.06 ± 0.17	0.219	2.57 ± 0.07	3.12 ± 0.07	<0.001	
Change	−0.80 ± 0.13	−0.32 ± 0.13	0.009	−0.68 ± 0.07	−0.31 ± 0.07	<0.001	

Abbreviations: DSE, diabetes support and education; ILI, intensive lifestyle intervention.

^a^ Interaction between having children in the home and treatment arm by ANCOVA using linear regression (continuous outcome) or logistic regression (binary outcome).

^b^ *x* ^2^ test.

^c^ Two‐sample t test.

^d^ Question assessed times exercised per week to a level of exertion that caused ‘sweat, shortness of breath or a thumping heart’.

## 4 DISCUSSION

This study found that among adults with diabetes, living in a home with children was associated with a decreased effect of an intensive behavioural weight loss intervention such that parents lost significantly less weight, and had less improvement in waist circumference, than nonparents. While the absolute difference in weight loss was small, parents lost 14.5% less weight than nonparents. The difference in weight loss between parents and nonparents was most pronounced among African American female participants. This is the first study to analyse the differential effects of a behavioural weight loss intervention between parents and nonparents. Parents were less likely to be in highest quartiles of attendance at intervention visits, and that attendance at intervention visits appears to be a mediator of the intervention effect. These findings of decreased attendance and weight loss support the overall hypothesis that parenting may make it challenging to respond favourably to weight loss interventions and highlight the need for more study in this area.

The effect of parenting on weight loss differed across racial subgroups, with a greater effect of parenting seen in African American participants and particularly among African American female participants. These findings were consistent with the 1‐year outcomes of the Look AHEAD trial, in which African American participants lost less weight than non‐Hispanic White and Hispanic/Latino participants.^30^ It is particularly important to examine this issue given persistent disparities in obesity among African American women^36^, ^37^ and among African American children.^38^ Because obesity in a parent increases the risk of obesity in a child,^8^ finding effective weight loss interventions for African American female parents may be important for reducing rates of obesity among African American persons in the United States. Overall, these findings call for more research to determine the mechanisms by which parenting influences the effects of weight loss interventions and why African American women may be most affected.

There was no difference in baseline BMI or age‐adjusted comorbidities by parenting status. It was hypothesized that parents would have lesser improvements in behaviour change than nonparents, and while most of the behavioural outcomes were worse in parents, none reach the level of significance. Parents reported an increase in caloric consumption of 21 kcal day^−1^, while nonparents reported a decrease of 42 kcal day^−1^. The difference was not significant in this analysis, but a difference of 63 kcal day^−1^ leads to an excess of 440 kcal week^−1^, or 6.5 lb in a year. The cumulative effect of many small differences in behaviour changes may be what contributed to the lower weight loss in parents compared with nonparents; however, it is also possible that there are unmeasured confounders associated with parenting that were not captured in this study.

Parental obesity is a significant risk factor for childhood obesity.^8^, ^9^, ^10^, ^11^ This increased risk to children's health, in addition to the risk to the parents' health, highlights the need for effective weight‐loss interventions for parents. A recent study showed that adults lost more weight when their children were supportive of their weight loss efforts,^39^ suggesting that parents may benefit from a family‐centred approach to weight loss. Research is urgently needed to design effective interventions tailored to parents, particularly those who are racial/ethnic minorities who have a higher risk of obesity‐related disease and may be disproportionally affected by the burdens of parenting.^40^ Such research should further address the complex interplay of social factors that may contribute to differential effects of parenting stress for racial/ethnic minorities and women.^41^ This may be particularly important when developing effective strategies to address significant racial/ethnic disparities in obesity and obesity‐related disease.^42^ Further, investigators should consider the burdens of parenting when designing and delivering weight management interventions so that they achieve the same efficacy regardless of parenting status.

This paper has several limitations. First, it is a retrospective analysis, and there are differences in baseline demographics between parents and nonparents that may not have been entirely accounted for by statistical adjustment. However, unadjusted and adjusted results were similar. Second, while referred to as parents and nonparents in this study, the relationship between the child(ren) in the home and the adult Look AHEAD participant is unknown. The average age of the participants may indicate that many are not parents. In addition, the exact age of the children in the home is unknown, and it is possible that parents with children of different ages will have different demands on their time and resources. To clarify effects from these potential confounders, future studies should specifically ascertain parenting relationships and ages of children. Additionally, as in most studies, eating and activity were assessed by self‐report. Accelerometry was assessed on a subset of participants in Look AHEAD, but we did not use this data because of the low number of parents. Finally, this study was conducted among adults aged 45 and older with overweight and obesity and Type 2 diabetes, so the results may not be generalizable to other populations.

## 5 CONCLUSIONS

In a clinical trial of an ILI for adults with Type 2 diabetes, participants with children living in the home had less improvement in weight and waist circumference than participants with no children living in the home. More research is needed to understand how having children in the home affects the ability of adults to achieve success in weight loss interventions and how these interventions can be tailored to meet their needs. Such research is necessary to improve the health of parents and ultimately prevent the intergenerational transfer of obesity to children.

## FUNDING

Dr. Bramante and Dr. Pilla were funded by the Behavioral Research in Heart and Vascular Disease Program Fellowship Training Program (T32HL007180‐41A1; PI: Hill‐Briggs, F.). The Look AHEAD Johns Hopkins site is funded under NIH/NIDDK grant U01DK057149 (site PI: Clark, JM). In 2019, Dr. Bramante was funded by the National Institutes of Health's National Center for Advancing Translational Sciences, grants KL2TR002492 and UL1TR002494. The content is solely the responsibility of the authors and does not necessarily represent the official views of the National Institutes of Health's National Center for Advancing Translational Sciences.

## CONFLICT OF INTEREST STATEMENT

The authors declare no conflicts of interest.
